# Supplementary material for: Graded titin cleavage progressively reduces tension and uncovers the source of A-band stability in contracting muscle
Source: eLife. 2020 Dec 24;9:e64107. doi: 10.7554/eLife.64107 (PMC7781594; doi:10.7554/eLife.64107)
Supplement: Transparent reporting form [file elife-64107-transrepform.docx]

***eLife’s* transparent reporting form**

We encourage authors to provide detailed information *within their submission* to facilitate the interpretation and replication of experiments. Authors can upload supporting documentation to indicate the use of appropriate reporting guidelines for health-related research (see [EQUATOR Network](http://www.equator-network.org/%20)), life science research (see the [BioSharing Information Resource](https://biosharing.org/)), or the [ARRIVE guidelines](http://www.plosbiology.org/article/info:doi/10.1371/journal.pbio.1000412) for reporting work involving animal research. Where applicable, authors should refer to any relevant reporting standards documents in this form.

If you have any questions, please consult our Journal Policies and/or contact us: [editorial@elifesciences.org](mailto:editorial@elifesciences.org).

**Sample-size estimation**

- You should state whether an appropriate sample size was computed when the study was being designed
- You should state the statistical method of sample size computation and any required assumptions
- If no explicit power analysis was used, you should describe how you decided what sample (replicate) size (number) to use

Please outline where this information can be found within the submission (e.g., sections or figure legends), or explain why this information doesn’t apply to your submission:

Studies were carried out using a number of 5-13 mice per genotype, depending on the type of experiment. Multiple fiber bundles could be prepared from a given muscle and a single animal. If we consider that a statistically meaningful change in mean values between treatments is >10%, using variation data from previous literature and our own published work on genetically modified mice, we made sure to have sample sizes that provided a power >80% and that enabled us to identify the main effects at a significance level of 0.05. If we found during experiments that data variability was larger than what we had expected, we reassessed sample needs accordingly.

The sample size is stated in all relevant figure legends.

**Replicates**

- You should report how often each experiment was performed
- You should include a definition of biological versus technical replication
- The data obtained should be provided and sufficient information should be provided to indicate the number of independent biological and/or technical replicates
- If you encountered any outliers, you should describe how these were handled
- Criteria for exclusion/inclusion of data should be clearly stated
- High-throughput sequence data should be uploaded before submission, with a private link for reviewers provided (these are available from both GEO and ArrayExpress)

Please outline where this information can be found within the submission (e.g., sections or figure legends), or explain why this information doesn’t apply to your submission:

If data are obtained from muscle fibers extracted from different mice, samples are independent from one another. However, fiber samples were also harvested from the same muscle, which is usual practice when trying to limit animal use. Measurements on these samples also represent biologically independent experiments. In any case, we made sure that we used > 3 different animals/genotype per experiment. In some mechanical analyses, multiple measurements were performed on the same sample; in such cases, the technical replicates are stated in the figure legend (together with the biological replicates), and this was accounted for by using a repeated-measures ANOVA design (sample ID as a random factor). No data were excluded. All other relevant information on the experimental and statistical design can be found in the Materials and Methods section (“Statistics”).

**Statistical reporting**

- Statistical analysis methods should be described and justified
- Raw data should be presented in figures whenever informative to do so (typically when N per group is less than 10)
- For each experiment, you should identify the statistical tests used, exact values of N, definitions of center, methods of multiple test correction, and dispersion and precision measures (e.g., mean, median, SD, SEM, confidence intervals; and, for the major substantive results, a measure of effect size (e.g., Pearson's r, Cohen's d)
- Report exact p-values wherever possible alongside the summary statistics and 95% confidence intervals. These should be reported for all key questions and not only when the p-value is less than 0.05.

Please outline where this information can be found within the submission (e.g., sections or figure legends), or explain why this information doesn’t apply to your submission:

A thorough description of statistical methods employed is included in Materials and Methods section. Sample sizes for each experiment are indicated in the figure legends. We added individual data points to all our graphs to demonstrate data variability. Furthermore, the source data with individual points and statistical results were prepared into a single file and submitted as a Supplemental dataset. We report the exact p-value in the main text only when it is close to 0.05. Otherwise, we indicate the p-value using asterisks, for p<0.05 (*), p<0.01 (**), and p<0.001 (***).

(For large datasets, or papers with a very large number of statistical tests, you may upload a single table file with tests, Ns, etc., with reference to sections in the manuscript.)

**Group allocation**

- Indicate how samples were allocated into experimental groups (in the case of clinical studies, please specify allocation to treatment method); if randomization was used, please also state if restricted randomization was applied
- Indicate if masking was used during group allocation, data collection and/or data analysis

Please outline where this information can be found within the submission (e.g., sections or figure legends), or explain why this information doesn’t apply to your submission:

Group allocation was created based on genotyping results. Genotyping for each mouse was conducted on ear punches using PCR. The relevant information on genotyping is provided in the Materials and Methods section (“Animal model and muscle preparation”). Genotypes are stated in all relevant figure legends or directly in the figures. Although animals purposely were not randomly selected from the pool of genotyped mice, we used any mice available that fit our age requirements for the study blindly (without prior visual assessment), unless deemed unusable by the attending veterinarian of our animal handling facility.

**Additional data files (“source data”)**

- We encourage you to upload relevant additional data files, such as numerical data that are represented as a graph in a figure, or as a summary table
- Where provided, these should be in the most useful format, and they can be uploaded as “Source data” files linked to a main figure or table
- Include model definition files including the full list of parameters used
- Include code used for data analysis (e.g., R, MatLab)
- Avoid stating that data files are “available upon request”

Please indicate the figures or tables for which source data files have been provided:

We provide source data files as supplemental information. Source data containing numerical data and statistical results (e.g. ANOVA and Student’s t-test) are provided as an Excel table for Figure 1C-F, Figure 2A-E, Figure 3B, Figure 4B and 4D, Figure 5B and 5D, Figure 6B and 6D, and Figure 7D. Uncropped gel images are shown in another source data file (pdf) for Figure 1E (Coomassie stained gel), Figure 1F (Western blot and corresponding Coomassie-stained membrane), Figure 4A (Coomassie stained gel), and Figure 6A (Coomassie stained gel). Our custom-made MATLAB code to calculate “fracture area” is at Github (<https://github.com/UKMPhysII/QuantitativeFractureCode>) with accession info provided in the main text. Further, we plan to publish this protocol in BioProtocols or a similar web-based repository.
